# New insights into *Escherichia coli* metabolism: carbon scavenging, acetate metabolism and carbon recycling responses during growth on glycerol

**DOI:** 10.1186/1475-2859-11-46

**Published:** 2012-07-04

**Authors:** Karla Martínez-Gómez, Noemí Flores, Héctor M Castañeda, Gabriel Martínez-Batallar, Georgina Hernández-Chávez, Octavio T Ramírez, Guillermo Gosset, Sergio Encarnación, Francisco Bolivar

**Affiliations:** 1 Departamento de Ingeniería Celular y Biocatálisis, Instituto de Biotecnología, Universidad Nacional Autónoma de México (UNAM), Apdo. Postal 510-3, Cuernavaca, Morelos, CP 62250, Mexico; 2 Departamento de Medicina Molecular y Bioprocesos, Instituto de Biotecnología, Universidad Nacional Autónoma de México (UNAM), Apdo. Postal 510-3, Cuernavaca, Morelos, CP 62250, Mexico; 3 Programa de Genómica Funcional de Procariotes, Centro de Ciencias Genómicas, Universidad Nacional Autónoma de México (UNAM), Apdo. Postal 565-A, Cuernavaca, Morelos, CP 62210, Mexico

## Abstract

**Background:**

Glycerol has enhanced its biotechnological importance since it is a byproduct of biodiesel synthesis. A study of *Escherichia coli* physiology during growth on glycerol was performed combining transcriptional-proteomic analysis as well as kinetic and stoichiometric evaluations in the strain JM101 and certain derivatives with important inactivated genes.

**Results:**

Transcriptional and proteomic analysis of metabolic central genes of strain JM101 growing on glycerol, revealed important changes not only in the synthesis of MglB, LamB and MalE proteins, but also in the overexpression of carbon scavenging genes: *lamB*, *malE*, *mglB*, *mglC*, *galP* and *glk* and some members of the RpoS regulon (*pfkA*, *pfkB*, *fbaA*, *fbaB*, *pgi, poxB*, *acs*, *actP* and *acnA*). Inactivation of *rpoS* had an important effect on stoichiometric parameters and growth adaptation on glycerol. The observed overexpression of *poxB*, *pta*, *acs* genes, glyoxylate shunt genes (*aceA, aceB*, *glcB* and *glcC*) and *actP*, suggested a possible carbon flux deviation into the PoxB, Acs and glyoxylate shunt. In this scenario acetate synthesized from pyruvate with PoxB was apparently reutilized via Acs and the glyoxylate shunt enzymes. In agreement, no acetate was detected when growing on glycerol, this strain was also capable of glycerol and acetate coutilization when growing in mineral media and derivatives carrying inactivated *poxB* or *pckA* genes, accumulated acetate. Tryptophanase A (TnaA) was synthesized at high levels and indole was produced by this enzyme, in strain JM101 growing on glycerol. Additionally, in the isogenic derivative with the inactivated *tnaA* gene, no indole was detected and acetate and lactate were accumulated. A high efficiency aromatic compounds production capability was detected in JM101 carrying pJLB*aroG*^*fbr*^*tktA*, when growing on glycerol, as compared to glucose.

**Conclusions:**

The overexpression of several carbon scavenging, acetate metabolism genes and the absence of acetate accumulation occurred in JM101 cultures growing on glycerol. To explain these results it is proposed that in addition to the glycolytic metabolism, a gluconeogenic carbon recycling process that involves acetate is occurring simultaneously in this strain when growing on glycerol. Carbon flux from glycerol can be efficiently redirected in JM101 strain into the aromatic pathway using appropriate tools.

## Background

**Figure 1:**
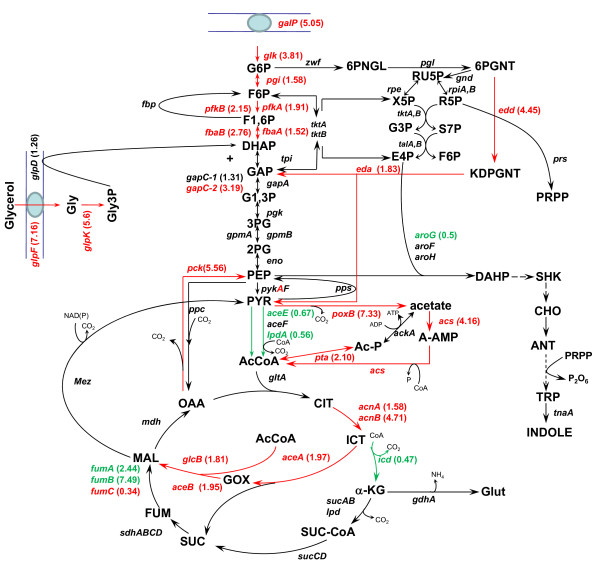
**Central metabolic reactions of strain JM101 growing on glycerol as the sole carbon source and relative gene transcription values as compared to the same strain grown on glucose.** Metabolites abbreviations: Gly, glycerol, Gly3P glycerol-3-phosphate; G, glucose; G6P, glucose-6-phosphate; F6P, fructose-6-phosphate; F1,6P, fructose-1,6-biphosphate; DHAP, dihydroxyacetone phosphate; G3P, glyceraldehyde 3-phosphate; G1,3P, 1,3-bisphosphoglycerate; 3PG, 3-phosphoglycerate; 2PG, 2-phophoglycerate; PEP, phosphoenolpyruvate; PYR, pyruvate; 6PGLN, 6-phosphoglucono-δ-lactone; 6PGNT, 6-phophogluconate; Ru5P, ribulose-5-phosphate; R5P, ribose-5-phosphate; Xu5P, xylulose-5-phosphate; S7P, seudoheptulose-7-phosphate; E4P, erythrose-4-phosphate; Ac-CoA, acetyl coenzyme A; Ac-P, acetyl phosphate; A-AMP, acetyl-AMP; CIT, citrate; ICT, isocitrate; GOX, glyoxylate; α-KG, α-ketoglutarate; SUC-CoA, succinyl-coenzyme A; SUC, succinate; FUM, fumarate; MAL, malate; OAA, oxaloacetate; KDPGNT, 2-keto-3-deoxy-D-gluconate-6-phosphate; PRPP, 5-phospho-D-ribosyl-α-1-pyrophosphate; DAHP, 3-deoxy-D-arabino-heptulosonate-7-phosphate; SHK, shikimate; CHO, chorismate; ANT, anthranilate; TRP, L-tryptophan. Genes in red: overexpressed. Genes in green: underexpressed. Genes in black: no change.

*Escherichia coli* is capable of utilizing several compounds as carbon sources. However, glucose is the preferred carbon source and its rapid utilization depends on the phosphoenolpyruvate: carbohydrate phosphotransferase system (PTS). PTS not only transports specific sugars but also in the absence of its substrates, stimulates, through adenylate cyclase (Cya), the production of *c*AMP which in turn activates the transcription of many *c*AMP-CRP dependent catabolic genes, including those involved in glycerol utilization [1]. Glycerol, an energy-poor carbon source, has enhanced its biotechnology importance as carbon source since it is a by-product of the biodiesel synthesis, whose production is expected to increase in the future [2-4]. A balanced aerobic growth on glycerol depends on three global regulators: *c*AMP-CRP as the principal inducer of the glycerol catabolic regulon (including *glpFglpK* and *glpD*); Cra (FruR) as regulator of some gluconeogenic genes, and ArcA as regulator of several central metabolic genes including the TCA cycle and others involved in respiration [1,5]. *E. coli* growing aerobically on glycerol incorporates this molecule into central metabolism as dihydroxyacetone phosphate (DHAP), a metabolite which can participate in both gluconeogenic and glycolytic processes (Figure 1) [6]. The expression of metabolic genes, in particular the overexpression of *pykApckA, gltAfumABCsdhmdh* and *acnA* genes and the downregulation of the *ackA* gene, has been reported for *E. coli* growing on glycerol [7]. Proteomic and enzymatic assay studies, in which cells were grown on a complete medium (Luria broth) plus glycerol, reported overexpression of the *fbp* gene and at lower levels *aceBA* operon [8]. However, studies on the carbon stress response of *E. coli* growing only on an energy-poor carbon source such as glycerol are scarce. It is know that *E. coli* displays carbon stress responses when growing under carbon source limitation similar to those encountered during stationary phase or glucose limited conditions in chemostats cultures [9,10], fed batch cultures [11], or in strains with limited glucose transport capabilities [12,13]. *E. coli* carbon stress response utilizes mechanisms which are part of the general stress response, and involve several changes in cellular physiology. This response can be a fast emergency strategy or a long-term program of adaptation to starvation [14]. The master regulator of the general stress response is the sigma factor RpoS, whose regulation is complex and involves transcriptional and posttranscriptional control mechanisms. For example, *rpoS* transcription is stimulated by downshifts in the specific growth rate (μ) [9,15,16]. Furthermore, a continuous reduction in μ, results in an inversely correlated increase in *rpoS* transcription (5 to 10- fold). In addition, when an abrupt cessation of growth occurs, as in response to sudden glucose starvation, *rpoS* transcription is also induced [17,18]. In strains lacking PTS which grow slowly on glucose as the sole carbon source, cells apparently sense low levels of carbon and induce *rpoS* transcription and several other genes involved in carbon scavenging, as well as a carbon (acetate) recycling mechanism [12,13,19-21]. Since *rpoS* expression is induced by different stresses, genes whose transcription are regulated by this sigma factor can be modulated by various coregulators such as Crl. Crl responds to indole as a signal molecule, increasing gene transcription of some genes by binding to RpoS. This in turn, stimulates RNA polymerase holoenzyme formation [22]. Carbon stress responses also involve amino acid starvation conditions in which ppGpp concentration increases rapidly to the millimolar range. When cells are growing under stress conditions, including slow growth on glucose, the expression of central metabolic genes is modified by altering the transcription of genes that redirect metabolism [12-14,19-21].

In this report some non described features detected in *E. coli* strain JM101 [23] grown in minimal medium on glycerol as the sole carbon source, are presented. Gene transcription levels and protein production patterns corresponding to a carbon stress response were detected. Overexpression of genes involved in the production and consumption of acetate that correlated with no detection of this metabolite in cultures of JM101 grown on glycerol, as well as the capability of acetate and glycerol coutilization in mineral media cultures were also detected in this strain, in agreement with a “carbon stress acetate recycling” response. Additionally, derivative strains with inactivated important genes such as *rpoS* and some members of the PEP-PYR node (*pckApoxBppcpykA* and *pykF*) were constructed and evaluated to clear the role of their coded proteins in the JM101 glycerol metabolism.

## Results and discussion

### Growth of strain **JM101** on different carbon sources

**Figure 2:**
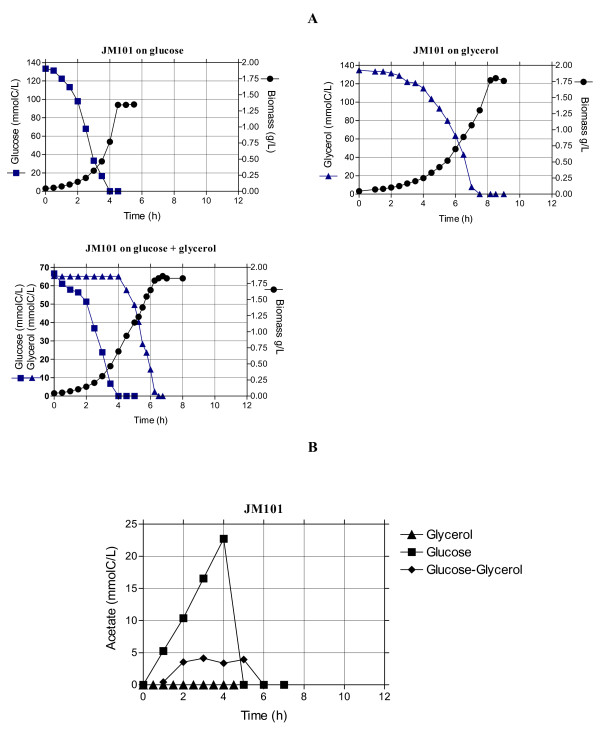
**A) Growth profiles and substrate utilization (mmolC/L) of strain JM101 grown on glucose or glycerol and in the mixture glucose plus glycerol.B**- Acetate levels (mmolC/L) of strain JM101 grown on glucose, or glycerol and on a mixture of glucose plus glycerol.

**Figure 3:**
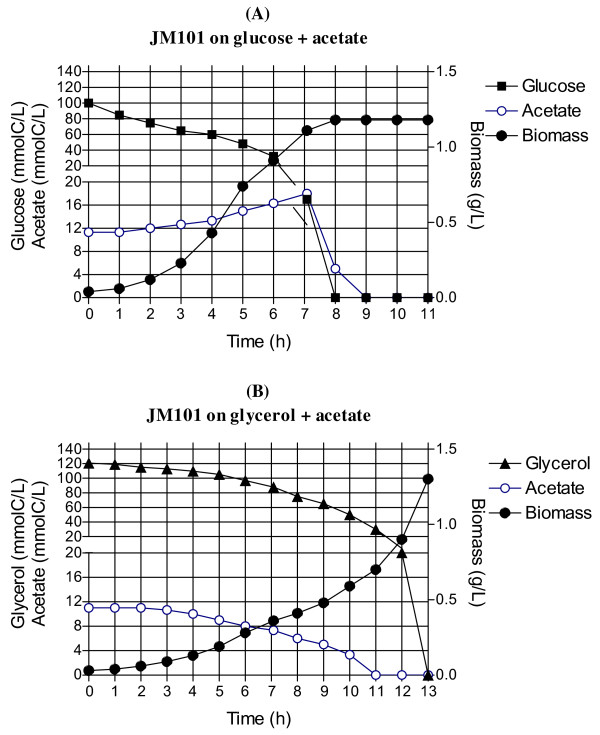
**Biomass and acetate consumption profiles (mmolC/L) of strain JM101 grown on a mixture of glucose plus acetate** (**A**) **and on a mixture of glycerol plus acetate** (**B**)**.**

Strain JM101 was grown on different carbon sources to compare its metabolic capacities. As shown in Table 1 and Figure 2, when this strain was grown on glucose, a high μ was obtained (0.69 h^−1^) and acetate was produced. When glycerol was used as the sole carbon source, its μ decreased to 0.49 h^−1^ and no acetate was detected (Table 1, Figure 2A). Low levels or no acetate production also have been detected in other *E. coli* strains grown on glycerol [7,8]. When both glucose and glycerol were present as carbon sources, as expected a diauxic response was obtained in JM101. Glucose was utilized first and glycerol was used after glucose had been completely depleted; however, less acetate was produced when this strain was grown on a mixture of these two carbon sources (Table 1, Figure 2B), suggesting that part of the acetate produced when growing on glucose as the sole carbon source, was utilized when both substrates were present in the medium. JM101 utilized glucose and accumulated acetate when grown with a mixture of glucose and acetate as carbon sources (Figure 3A). In contrast, when a mixture of glycerol and acetate was used, JM101 coutilized both carbon sources (Table 1, Figure 3B).

**Table 1:** Specific growth rates (μ) and stoichiometric parameters of strain JM101 grown on single or mixtures of carbon sources

**Condition**	**μ (h**^**−1**^**)**	**Y**_**x/s**_**(g/mmolC)**	**qs (mmolC/g**_**dcw**_**h)**	**mmolC of acetateproduced (+) or consumed (−)**
Glucose	0.69	0.013	51.8	+28.2
Glycerol	0.49	0.014	34.3	Not detected
Glucose + glycerol	0.72 (0.45)	0.017(0.006)	43.1	+4.1
Glycerol + acetate	0.43	0.011	39.5	−11.0
Glucose + acetate	0.72(0.1)	0.013(0.017)	55.4(6.55)	+6.0

Mean values from at least three independent cultures are presented. Differences between values in these experiments were <10%. The numbers in parentheses indicate data for the specific strain in those stages in which only the remaining carbon source was available.

### Grown on glycerol of different JM101 derivatives

The absence of acetate production in strain JM101 growing on glycerol suggested that in this strain production and consumption of acetate occurred simultaneously, as has been reported in strains derived from JM101 lacking PTS that grow slowly on glucose. In these derivatives where the glycolytic metabolism is apparently functioning, the gluconeogenic metabolism is induced and glucose and acetate are utilized simultaneously [12,13,19,21,24]. These results indicate that growth on glycerol of strain JM101 could activate some important gluconeogenic genes like *poxB* and *pckA* involved in carbon (acetate) recycling. Therefore, JM101 derivatives were constructed with inactivated key genes including *rpoSpoxB* and *pckA* (see Methods), in order to evaluate the role of their coded proteins in the glycerol fermentation, testing the effects on μ, q_s_ (glycerol specific rate consumption), and Yx/s (biomass/substrate yield), when growing in mineral medium with glycerol or glucose as the sole carbon source.

The JM101Δ*rpoS* derivative had a two-hour delay for growing in mineral media with glycerol (data not shown) and once adapted, the μ was 10% slower than the parental strain (Table 2A). This delay was not observed in the derivative growing with glucose (data not shown), which had a lower q_s_, (28%) and a higher Y_x/s_ (23%) as compared to strain JM101 (Table 2B). RpoS is not directly involved in the expression of the genes that incorporate glycerol in the central metabolism (*glpFglpD* and *glpK*); however, its relation with *glpD* is apparently indirect through ArcAB. Some reports argue a feedback loop, RpoS-mediated stationary phase induction of the *arcA* gene [25]. It has been reported that RpoS regulates the expression of *poxB* and other catabolic genes involved in carbon stress responses [12,20,26]. Therefore, it appears that RpoS, the master regulator of the stress response, is important for adapting and maintaining an adequate balanced growth on glycerol as the sole carbon source in mineral media, as compared to the strain grown on glucose (Tables 2A and B).

**Table 2:** Specific growth rates (μ) and stoichiometric parameters of strain JM101 and derivative mutants grown on glycerol (A) and glucose as carbon source (B)

**A)**				
**Strain**	**μ (h**^**-1**^**)**	**Y**_**x/s**_**(g/mmolC)**	**q**_**s**_**(mmolC/gdcwh)**	**Acetate (g/L)**
JM101	0.49 (+/–0.02)	0.014 (+/–0.001)	34.6 (+/–1.90)	No detected
JM101Δ*pckA*	0.45 (+/–0.03)	0.014 (+/–0.001)	33.3 (+/–4.56)	0.12 (+/–0.01)
JM101Δ*poxB*	0.44 (+/–0.01)	0.014 (+/–0.001)	35.5 (+/–2.14)	0.12 (+/–0.01)
JM101Δ*ppc*	No growth			
JM101Δ*pykA*	0.46 (+/–0.02)	0.015 (+/–0.001)	30.3 (+/–0.12)	Not detected
JM101Δ*pykF*	0.46 (+/–0.02)	0.014(+/–0.002)	31.4 (+/–1.00)	Not detected
JM101Δ*rpoS*	0.44 (+/–0.02)	0.018 (+/–0.002)	24.8 (+/–2.57)	Not detected
**B)**				
**Strain**	**μ (h**^**−1**^**)**	**Y**_**x/s**_**(g/mmolC)**	**q**_**s**_**(mmolC/gdcwh)**	**Acetate (g/L)**
JM101	0.69 (+/–0.03)	0.013 (+/–0.001)	51.8 (+/–2.22)	0.5 (+/–0.01)
JM101Δ*pckA*	0.62 (+/–0.09)	0.023 (+/–0.011)	50.7 (+/–16.82)	0.7 (+/–0.02)
JM101Δ*poxB*	0.68 (+/–0.04)	0.014 (+/–0.002)	50.7 (+/–2.50)	0.4 (+/–0.02)
JM101Δ*ppc*	No growth			
JM101Δ*pykA*	No determinated	No determinated	No determinated	
JM101Δ*pykF*	No determinated	No determinated	No determinated	
JM101Δ*rpoS*	0.71 (+/–0.01)	0.013 (+/–0.004)	54.5 (+/–8.34)	0.5 (+/–0.01)

The JM101Δ*poxB* derivative had the same q_s_ and Y_x/s_ as the parental strain but its μ was also reduced 10% and accumulated acetate in the culture, when growing on glycerol (Table 2A). This result can be explained in two ways: *poxB* inactivation could be responsible for *acs* underexpression and/or could indicate that utilization of PYR through PoxB to produce acetate and the concomitant reduction of quinones at the membrane [18], could be necessary for the complete induction of the glyoxylate shunt (Figure 1). Additional evidence supporting PoxB involvement in acetate utilization when glycerol is utilized as unique carbon source, is presented in the next section, where the overexpression of *poxBacsaceBA* genes is presented and discussed. Nevertheless it is important to notice that the JM101Δ*rpoS* derivative did not accumulate acetate which is in fact produced in the JM101Δ*poxB* derivative. RpoS as mentioned is involved in the expression of carbon stress genes including *poxB*. Therefore, it appears that as has been proposed *poxB* and other members of the RpoS regulon could be transcribed also by RpoD, the vegetative sigma factor, in certain grown conditions, including slow grown on glucose [12,18-20] and in agreement with these results, maybe in glycerol fermentation. In the absence of RpoS, *poxB* could be transcribed by RpoD, explaining the absence of acetate in JM101Δ*rpoS*[20]. In JM101Δ*poxB* acetate is produced indicating the necessity of PoxB in acetate recycling metabolism. An alternatively not exclusive explanation for the absence of acetate in JM101Δ*rpoS* is that in this derivative, a lower glycerol specific consumption occurred which could be responsible of less PEP production which is sensed as a low carbon flux that in turn induces carbon reutilization.

The JM101Δ*pckA* derivative had 2 h adaptation time for growing in mineral media as compared to the parental strain JM101 (data not shown), accumulated acetate during fermentation and the μ was reduced about 10% when growing on glycerol. However, the q_s_ and Y_x/s_ values were not substantially affected in this mutant strain (Table 2A). PckA appears to be important for glycerol utilization and also for completing a gluconeogenic cycle to prepare strain JM101 to grow in a gluconeogenic substrate such as acetate. Production of acetate in this mutant also indicates that carbon flux through PckA could be important to maintain ATP/ADP levels, since it has been reported that overexpression of *pckA* enhances ATP intracellular levels [27] (Figure 1). This proposal is supported by transcription results presented in the next section, where overexpression of *pckA* is presented and discussed.

The derivative carrying the *ppc* gene inactivated was incapable of growing on glycerol or glucose as the sole carbon sources (Tables 2A–B), indicating that Ppc is essential to grow in these two carbon sources, and cannot be replaced by the Pyk enzymes (Figure 1) and PEP has to be converted directly into OAA using Ppc to allow growth on glucose or glycerol as sole carbon sources. Similar results have been reported by others [28-31].

Derivatives carrying *pykA* or *pykF* inactivated genes did not substantially modify the specific growth rates as compared to the parental strain, when growing on glycerol as the sole carbon source (Table 2A).

### Differential transcription of genes and coded proteins production in the strain JM101 grown on glycerol, as compared to glucose

#### *Glycerol uptake*

Glycerol is converted into DHAP by a process of uptake (GlpF), phosphorylation (GlpK), and dehydrogenation (GlpD) (Figure 1). As expected, *glpF* and *glpK* were overexpressed in strain JM101 grown on glycerol (Table 3). In agreement, the protein products of these genes, GlpK (16.18X) and GlpD (5.18X) -involved in the transformation of glycerol into glycerol-3-phosphate (Gly3P) and this metabolite into DHAP, respectively- were overproduced in this strain (Table 4). Surprisingly, the expression of *glpD* was not substantially modified (Table 3, Additional file 1). Interestingly, the transcription of *glpF* and *glpK* is regulated by CRP and GlpR, whereas the expression of *glpD* is regulated by CRP, GlpR and ArcA, as proposed elsewhere [1,5].

**Table 3:** Relative transcription levels determined by RT-qPCR of several group of genes from strain JM101 on glycerol as the only carbon source

**Glycolysis**	**Gluconeogenic, anaplerotic and glyoxylate shunt**	**TCA cycle**
*aceE* 0.67 +/- 0.17	*aceA* 1.97 +/- 0.39	*acnA* 1.58+/- 0.26
*aceF* 0.80 +/- 0.22	*aceB* 1.95 +/- 0.06	*acnB* 4.71+/- 0.47
*eno* 0.86 +/- 0.12	*ackA* 1.49 +/- 0.28	*fumA* 2.44+/- 0.45
*fbaA* 1.52 +/- 0.09	*acs* 4.16 +/- 0.34	*fumB* 7.49+/- 0.40
*fbaB* 2.76 +/- 0.22	*fbp* 1.51 +/- 0.10	*fumC* 0.34+/- 0.10
*gapA* 0.72 +/- 0.06	*glcB* 1.81 +/- 0.56	*gltA* 0.76+/- 0.02
*gapC-1* 1.31 +/- 0.10	*glcC* 1.81 +/- 0.28	*icd* 0.47+/- 0.10
*gapC-2* 3.19 +/- 0.67	*maeB* 1.23 +/- 0.01	*mdh* 0.65+/- 0.10
*glk* 3.81 +/- 0.14	*pckA* 5.56 +/- 1.77	*sdhA* 1.04+/- 0.01
*gpmA* 0.90 +/- 0.09	*poxB* 7.33 +/- 0.56	*sdhB* 1.37+/- 0.42
*lpdA* 0.56 +/- 0.05	*ppc* 1.11 +/- 0.14	*sdhC* 0.92+/- 0.05
*pfkA* 1.91 +/- 0.22	*pta* 2.10 +/- 0.23	*sdhD* 1.13+/- 0.21
*pfkB* 2.15 +/- 0.01	*ppsA* 1.26 +/- 0.22	*sucA* 0.81+/- 0.13
*pgi* 1.58 +/- 0.37	*sfcA* 2.05 +/- 0.34	*sucB* 0.80+/- 0.23
*pgk* 1.08 +/- 0.13		*sucC* 0.79+/- 0.07
*pykA* 1.77 +/- 0.10		*sucD* 1.36+/- 0.13
*pykF* 0.98 +/- 0.09		
*tpi* 1.36 +/- 0.10		
**Pentose phosphate Entner-Doudoroff**	**Glycerol uptake**	***mal/lam*and*mgl/gal*regulons**
*eda* 1.83+/-0.29	*glpF 7.16* +/- *0.15*	*galP* 5.05+/-0.26
*edd* 4.45+/-0.29	*glpK 5.60* +/- *0.87*	*galR* 0.38+/-0.13
*gnd* 0.80+/-0.00	*glpD 1.26* +/- *0.25*	*galS* 0.57+/-0.01
*talA* 1.64+/-0.47	*glpR 2.40* +/- *0.06*	*galT* 0.49+/-0.00
*talB* 0.60+/-0.01		*lamB* 1.36+/-0.30
*tktA* 1.22+/-0.06		*mglB* 5.05+/-0.58
*tktB* 0.70+/-0.11		*mglC* 2.28+/-0.12
*zwf* 1.34+/-0.04		*malE* 1.77+/-0.20
		*pgm* 2.37+/-0.62
**Aromatic pathway**	**Respiration, transhydrogenases**	**Transporters/ porins**
*aroA* 0.81 +/- 0.03	*frdA* 1.58+/-0.15	*actP 13.49*+/-*1.31*
*aroB* 1.98 +/- 0.59	*frdB* 1.73+/-0.11	*aroP 1.61*+/-*0.00*
*aroC* 1.34 +/- 0.02	*frdC* 4.68+/-0.21	*shiA 0.75*+/-*0.02*		
*aroD* 3.29 +/- 0.07	*frdD* 2.01+/-0.34	*ompC 0.17* +/- *0.01*		
*aroE* 5.47 +/- 0.16	*pntA* 0.69+/-0.03	*ompF 1.17* +/- *0.21*		
*aroF* 1.32 +/- 0.08	*udhA* 4.89+/-0.88			
*aroG* 0.50 +/- 0.13				
*aroH* 1.36 +/- 0.13				
*aroK* 1.01 +/- 0.07				
*aroL* 2.48 +/- 0.09				
**Regulators**				
*arcA 1.25*+/-*0.29*				
*arcB 4.45*+/-*0.56*				
*creB 3.19*+/-*0.55*				
*creC 6.27*+/-*1.69*				
*crp 1.71*+/-*0.12*				
*csrA 1.03*+/-*0.14*				
*csrB 0.48*+/-*0.10*				
*cyaA 1.64*+/-*0.24*				
*fadR 1.22*+/-*0.31*				
*fruR 3.00*+/-*0.20*				
*iclR 0.29*+/-*0.10*				
*ihfA 1.15*+/-*0.25*				
*mlc 1.18*+/-*0.15*				
*pdhR 0.57*+/-*0.19*				
*ptsG 0.14* +/-*0.02*				
*rpoS 1.04*+/-*0.22*				

Transcription levels of the measured genes of strain JM101 grown on glucose were used as controls to normalize (as one) the data using the RT-qPCR value for the corresponding gene. Results presented are the averages of at least three independent measurements of the RT-qPCR expression values for each gene. Expression levels are presented as 2^−ΔΔCT^ values (see Methods).

**Table 4:** Proteins differentially produced in strain JM101 grown on glycerol, as compared to the production on glucose

**Spot No.**	**Gene name**	**Protein description**	**Mw**	**pI**	**Ratio IODglycerol/IODglucose**
B11	*argG*	Argininosuccinate synthase	50038	5.23	0.37
58	*cdd*	Cytidine deaminase	31805	5.42	6.97
51	*fumA*	Fumarate hydratase class I	60774	6.11	2.38
57	*mglB*	D-galactose-binding periplasmic protein	35690	5.68	81.03
11	*glpD*	Aerobic glycerol-3-phosphate dehydrogenase	56886	6.97	5.18
D11	*glpK*	Glycerol kinase	56480	5.36	16.18
75	*glnA*	Glutamine synthetase	52099	5.26	0.31
60	*argT*	Lysine-arginine-ornithine-binding periplasmic protein	28088	5.62	2.57
P10	*ompC*	Outer membrane protein C	40343	4.58	0.32
31	*malE*	Maltose-binding periplasmic protein	43360	5.53	7.07
27	*lamB*	Maltoporin	49995	4.81	6.94
50	*pckA*	Phosphoenolpyruvate carboxykinase [ATP]	59891	5.46	9.17
73	*ptsI*	Phosphoenolpyruvate-protein phosphotransferase	63750	4.78	0.23
D10	*deoD*	Purine nucleoside phosphorylase deoD-type	26161	5.42	2.08
63	*gatY*	D-tagatose-1,6-bisphosphate aldolase subunit	31021	5.87	55.83
62	*udp*	Uridine phosphorylase	27313	5.81	7.41
K10	*tnaA*	Tryptophanase	53119	5.88	17.03
72	*typA*	GTP-binding protein TypA/BipA	67542	5.16	0.44

Spot intensities were quantified using PD Quest software 8.0.1 on three experiments. Only reproducible phenotypes, with a Student’s *t* test value p ≤ 0.05 are shown. Additional file 3 contains other important proteomic parameters.

*pI*: Isoelectric point.

*Mw*: Molecular weight.

*IOD*: Integrated optical density.

#### *Glucose and other carbohydrate scavengers*

Strain JM101 grown on glycerol overexpressed, as compared to the expression values grown on glucose, several genes of the *mal*/*lam* and *mgl*/*gal* regulons: *lamBmalEmglBmglCgalP, glk* and *pgm* (Table 3, Additional file 1). As shown in Table 4, the products of some of these genes, MglB (81.02X), LamB (6.94X) and MalE (7.06X) were produced at higher concentrations in the strain grown on glycerol. The *mal/lam* system in *E. coli* contains genes involved in transport and catabolism of maltose or maltodextrins [32-34] and the *mgl*/*gal* system contains genes related to the transport (*galP* and *mglBAC*) and amphibolic utilization (*galETKM*) of D-galactose [34-36]. In *E. coli*, the induction of both regulons has been reported as a response to growth at low glucose-carbon conditions such as those encountered in chemostats at low dilution rates or in starving cells [9,10,34-37]. Also, strains with limited glucose transport capacity growing slowly on this carbohydrate, such as those lacking PTS, overexpress the *mal*/*lam* and *mgl*/*gal* regulons, in a “nutrient scavenging stress response” [10,13,19]. In these scenarios, increased expression levels of these regulons have been reported, since high levels of cAMP-CRP are detected and the synthesis of maltotriose (*mal*/*lam*) and D-galactose (*mgl/gal*) as autoinducers are produced [1,9,10,34,38]. These results are in agreement with those reported by Liu et al. (2005) which indicate that poor carbon sources like glycerol, develops in *E. coli* a “carbon source foraging strategy” [37]. In strain JM101 grown on glycerol as the sole carbon source, high levels of *c*AMP and glycogen synthesis are expected, as well as glycogen degradation to maltodextrins-glucose and D-galactose. In this strain, *galTgalR* and *galS* genes were apparently slightly underexpressed, whereas the *galP* gene was overexpressed (Table 3). Furthermore, the *gal* regulon genes are differentially regulated by cAMP-CRP and D-galactose, and in the absence of D-galactose the expression of the *mglBAC* operon can be activated by cAMP-CRP, whereas the transcription of *galP* gene is strongly repressed [38,39]. The inactivation of *galP* did not modify the μ of JM101Δ*galP* grown on glycerol (data not shown), indicating that GalP is not playing an important role in glycerol transport. The *malE* gene and the *mglBAC* operon are transcribed by RpoS. In *E. colirpoS* is induced when cells are growing under stress conditions such as carbon, phosphorus, nitrogen or amino acid starvation. Therefore, RpoS apparently replaces, at least partially, the vegetative sigma factor RpoD in the transcription of certain genes when growing in glucose limited conditions [12,19,20,37,40]. It has been reported that the regulatory protein Crl increases RpoS activity by direct interaction with the RpoS holoenzyme [41]. In addition, the MalE protein is produced at low levels in *E. coli crl* or *crl*/*rpoS* mutants, as compared to the wild type strain [41]. Therefore, it is possible that in JM101 growing on glycerol, RpoS-Crl could upregulate the expression of some members of these regulons. In JM101 grown on glycerol, the OmpC porin was produced at lower levels as compared to glucose (Table 4). The *ompC* gene, which was underexpressed in this condition (Table 3), has a complex regulation including protein regulators (OmpR, CpxR, Lrp, IHF and EnvY) and small regulatory RNAs (*micCrseX* and *rybB*) that respond to different signals [42-46]. In JM101, the low expression levels of *ompC* and the low production of OmpC were apparently coordinated with the high production of LamB, which appears to be the principal porin when this strain was grown on this carbon source (Tables 3 and 4, Additional file 1). Since *lamB* expression level is increased when nutrients are scarce, it has been proposed that LamB can transport a wide variety of sugars such as glucose, lactose, arabinose, and even glycerol [10,13,19,47]. During the growth of JM101 on glycerol, it is possible that sensing of low nutrient availability or low osmolarity, compared to the growth on glucose, could be part of the signals involved in the reduced production of OmpC.

### Central metabolism genes

#### Glycolysis

**Figure 4:**
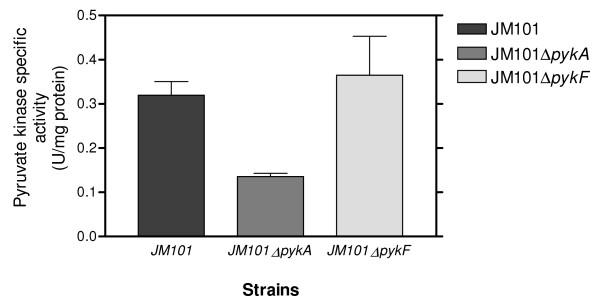
**Specific activities of the pyruvate kinases in strains JM101**Δ***pykA*and JM101**Δ***pykF*respectively, grown on glycerol, as compared to the strain JM101 grown on glycerol.**

The *glk* gene, which codes for glucokinase (Glk), was overexpressed in JM101 grown on glycerol (Table 3). Glk phosphorylates glucose into G6P during its transport from the periplasm to the cytoplasm by GalP or MlgABC [6,12,13,48]. *glk* carries a putative RpoS promoter [20] and is apparently repressed by Cra (FruR) [49]. The *pgi* gene was also slightly overexpressed in this strain (Table 3); as well as in strains lacking PTS growing slowly on glucose as the only carbon source [13]. Pgi is involved in the reversible transformation of G6P into fructose-6-phosphate (F6P) (Figure 1). In the upper part of the glycolytic pathway, *pfkA* and *pfkB* encoding for phosphofrukinase I and II, respectively, were overexpressed (Table 3, Additional file 1). PfkA and PfkB convert F6P to fructose-1,6P (F1,6P) consuming one ATP molecule (Figure 1). These proteins are an important control point of the glycolytic flux. The *pfkA* gene is positively regulated by CsrA [49] and has a putative RpoS promoter [20,41]. Additionally, *E. coli* strains inactivated in the *crl* or *rpoS* genes produced lower levels of PfkA as compared to the parental strain, suggesting that this gene is part of the RpoS regulon [41]. The expression of *pfkB* is repressed by CsrA [49] and this gene has a putative promoter that could be transcribed by RpoS [20,50,51]. Interestingly GatY, a protein that is part of the galactitol degradation pathway, was produced at very high levels (55.83X) in this strain grown on glycerol, as compared to those on glucose (Table 4). GatY is a reversible enzyme that catalyzes the synthesis of tagatose-1,6 phosphate from DHAP and GA3P; it is regulated by CRP, ArcA and GatR. GatY overproduction apparently occurs as a growth response on glucose-limited conditions such as those found in fed batch cultures [52]. The transcripts of the gluconeogenic *fbaA* and *fbaB* genes, whose coded proteins are involved in the reversible transformation of DHAP and glyceraldehyde 3-phosphate (G3P) into F1,6P, were overexpressed in JM101 (Table 3). This is an important gluconeogenic step for glycerol metabolism (see below). DHAP can be transformed into G3P by TpiA (Figure 1). DAHP and G3P can be transformed into F1,6P through FbaA and FbaB. Additionally, G3P is a key metabolite that can be utilized in several pathways including the glycolytic and pentose pathways (see below). This compound is transformed by GapA into G1,3P, which in turn is transformed into PEP through the participation of several glycolytic enzymes (Figure 1). Interestingly, the expression levels of *tpiAgapAgpmA* and *pgk* were not substantially modified in JM101 grown on glycerol as compared to the values obtained for the growth on glucose (Table 3). *E. coli* has two pyruvate kinases catalyzing the conversion of PEP into PYR coupled to the synthesis of ATP. These isoenzymes, PykA and PykF, are coded by the *pykA* and *pykF* genes, respectively. This reaction is the second point of flux control in the glycolytic pathway (Figure 1). Strain JM101 slightly overexpressed the *pykA* gene (Table 3). This data is in agreement with previous reports of other *E.coli* strains grown on glycerol as the only carbon source [7,8]. The *pykA* and *pykF* genes have putative promoters that could be transcribed by RpoS, and *pykF* is negatively regulated by Cra [20,53]. PykF is activated by F1,6P, whereas PykA is modulated by AMP and sugars of the pentose-phosphate pathway. Consequently, in conditions of low F1,6P levels as expected in cells grown on glycerol, PykA is probably the enzyme mainly utilized for growth [7]. As mentioned, single inactivation derivatives were constructed in the two genes coding for these pyruvate kinases and their growth on glycerol was evaluated. No significant differences were found in the μ of these mutants as compared to the parental strain (Table 2A). However, the total specific activity of pyruvate kinase was lower in the *pykA* mutant strain as compared to the strain with *pykF* inactivated or the parental strain (Figure 4). These results suggest that PykA was produced preferentially when JM101 was grown on glycerol and, therefore, it is apparently responsible for the main pyruvate kinase activity *in vivo*. Finally, PYR is utilized by the pyruvate dehydrogenase (Pdh) enzyme for the synthesis of AcCoA. The glycolytic genes, whose coded products are involved in this transformation (*aceEaceF* and *lpdA*), were slightly underexpressed in JM101 grown on glycerol, as compared to the growth on glucose (Table 3). These results clearly indicate that the glycolytic metabolism is functional in JM101 and are in agreement with a carbon flux deviation through PoxB for acetate production that has been proposed for slow-growing JM101 derivatives such as PB11 that lacks PTS [12,13,19,20], and in agreement with the overexpression of *poxBacs*, and the glyoxylate shunt genes in JM101 in these growing conditions [12,13,19,20] (see below).

#### The pentose pathway, Entner-Doudoroff and gluconeogenic metabolism in the upper glycolytic metabolism

Several gluconeogenic reactions are involved in the upper glucose metabolism pathway to allow gluconeogenic metabolism and the synthesis of G6P from glycerol (Figure 1). Glycerol is incorporated as DAHP and this metabolite and G3P are transformed into F1,6P. This compound is converted to F6P, and later transformed into G6P. The *fbaA*, *fbaB* and *fbp* genes involved in these gluconeogenic steps were overexpressed in JM101 (Table 3, Additional file 1). Interestingly, only *edd* and *eda* corresponding to the Entner-Doudoroff pathway were overexpressed in the pentose pathway (Table 3). The product of this last gene is involved in the synthesis of G3P and PYR from 2-keto-3-deoxy-D-gluconate-6-phosphate (KDPGNT) (Figure 1).

#### Acetate pathways, anaplerotic and other gluconeogenic genes

**Figure 5:**
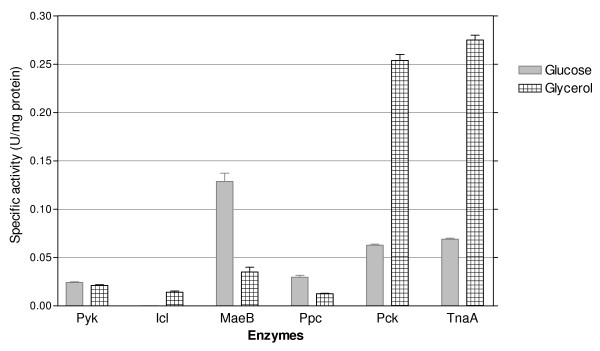
Specific activities of certain central metabolic enzymes of strain JM101 grown on glycerol, as compared to the same specific activities of the strain grown on glucose.

The *poxBacs, actP* and *pta* genes are involved in transport, production and consumption of acetyl-phosphate (Ac-P) and acetate (Figure 1). *poxBacs* and *actP* are overexpressed in conditions of glucose starvation and are part of the RpoS regulon [12,25,26,54]. These genes were also overexpressed in JM101 grown on glycerol. Additionally, the overexpression of *aceBaceA* and *glcB* in these growing conditions suggests the induction of the glyoxylate pathway (Table 3, Additional file 1). Since no acetate was detected during the growth of JM101 on glycerol, it is possible that a fraction of the acetate was synthesized via PoxB and in turn, this metabolite was transformed into AcCoA by Acs, apparently inducing a gluconeogenic response utilization of acetate by the glyoxylate shunt enzymes, as has been proposed for strain PB11 [11,18]. To confirm this hypothesis, strain JM101 with an inactivated *poxB* gene (JM101Δ*poxB*) grown on glycerol was evaluated. This derivative accumulated acetate during fermentation and its μ was reduced 10% as compared to the parental strain (Table 2A), indicating a role of PoxB in acetate metabolism growing on glycerol as the carbon source. In agreement, the isocitrate lyase specific activity (Icl or AceA, one of the glyoxylate shunt enzyme) was detected in JM101 grown on glycerol but not on glucose as the only carbon source (Figures 1 and 5). The *actP* gene, which was also overexpressed is part of the *acs* operon, is positively regulated by CRP and can be transcribed by 54, 70 and 38 (RpoS) sigma factors [12,20,24,55]. Therefore, it appears that since glycerol is a poor carbon source, part of the response involved in carbon scavenging (including acetate reutilization or recycling) is activated in this strain when growing on glycerol. In agreement, genes involved in the synthesis of pyruvate dehydrogenase were slightly underexpressed in these growing conditions, indicating that part of the carbon flux is apparently directed and recycled via PoxB/Acs and the glyoxylate shunt [12,13,20,37,56,57]. The *pckA* gene was also highly overexpressed and its coded protein was synthesized at high levels in JM101 (Tables 3 and 4); consequently, the PckA specific activity was higher on glycerol as compared to glucose (Figure 5). The overexpression of this gene in other *E. coli* strains grown on glycerol has been previously reported [8,58]. The *pckA* gene is regulated by Cra, and PckA synthesizes PEP from oxalacetate (OAA), with the production of ATP and CO_2_. OAA is an indispensable metabolite for the TCA cycle regeneration and it is precursor of aspartate (Figure 1). It appears that when glycerol is used as the sole carbon source and the proposed acetate-glyoxylate shunt and carbon recycling program is running, PckA is apparently involved in the gluconeogenic cycle. In accordance are the facts that MaeB is an enzyme that utilizes NADPH_2_ for converting MAL to PYR, the *maeB* gene was not upregulated and the specific activity of this enzyme was lower on glycerol as compared to glucose, indicating a role of PckA in the gluconeogenic pathway, in these growing conditions (Figures 1 and 5). In agreement, *pckA* inactivation as mentioned decreased 10% the μ of the derivative strain as compared to the parental strain (Table 2A). Interestingly and in agreement with this acetate recycling proposal, when JM101 was grown on a mixture of glycerol plus acetate, the μ of this strain was not enhanced; however, acetate was coutilized with glycerol (Table 1 and Figures 2 and 3).

#### TCA cycle

The *acnA* and *acnB* genes were overexpressed in strain JM101 grown on glycerol as compared to glucose (Table 3, Additional file 1). These genes code for aconitases A and B respectively, involved in the synthesis of isocitrate (ICT) from citrate (CIT) (Figure 1). The *acnA* gene is positively regulated by Crp, SoxRS, FNR and repressed by ArcA [59], whereas *acnB* is positively regulated by CRP and negatively regulated by ArcA, FruR and Fis. AcnB appears to be the main catabolic enzyme in *E. coli* and AcnA is apparently used in nutritional or oxidative stress [60]. Moreover, overexpression of *acnB* has been detected in strains growing on acetate as the only carbon source [60]. Importantly, the transcription level of *icd* was lower in JM101 grown on glycerol (Table 3). Icd is regulated by phospho/dephosphorylation and synthesizes α-KG and NADPH_2_ from ICT. α-KG is an important metabolite for glutamate biosynthesis (Figure 1). Therefore, a possible flux reduction through Icd is in agreement with the proposed of a reduction of the flux in the lower section of the TCA cycle in JM101. However, this proposal is not in agreement with previous reports of carbon fluxes in other *E. coli* strains where apparently carbon flux through Icd is slightly enhanced when glycerol is used as carbon source as compared to glucose [7]. The *fumA* and *fumB* genes were also overexpressed in JM101, whereas *fumC* was underexpressed (Table 3); in agreement, FumA was synthesized at higher levels in this strain (Table 4). The *fumA* gene transcription is positively regulated by Crp and repressed by ArcA and its expression is predominant under aerobic conditions. The *fumB* gene overexpression was unexpected since this gene is synthesized preferentially on anaerobic conditions. Interestingly, in JM101 grown on glycerol, the *frdABCD* operon (encoding the anaerobic fumarate reductase complex) was also overexpressed (Table 3). The *fumB* gene is positively regulated by FNR, ArcA, Crp and Fur and negatively by Fis and NarL. The *frdABCD* operon is also positively regulated by FNR and negatively by NarL. Remarkably, the two-component signal system DcuS-DcuR induces transcription of the *dcuB-fumB* operon, the *frdABCD* operon, and *dctA* (C4 compounds transporter) in response to external C4 compounds (SUC, FUM, MAL, and aspartate) [61,62]. Importantly, JM101 grown on glycerol enhanced its μ when small quantities (0.005 g/L) of SUC or MAL were included in the medium (Table 5), suggesting a lower C4 compounds production when growing on glycerol. From these results it can be concluded that at least another signal is apparently involved in the overexpression of the *frd* operon and *fumB* in this strain grown on glycerol. There was no substantial difference in the expression of the *sdhCDAB* and *sucABCD* operons in JM101 grown on glycerol as compared to glucose (Table 3). However, the transcription of *lpdA* was underexpressed. Among other roles, the product of this gene is a component of the SucABCD complex involved in the transformation of α-KG into SUC. These results suggest that a lower carbon flux is probably present in the lower section of the TCA cycle including the Icd and SucA enzymes in strain JM101 (Figure 1). These observations are in agreement with the proposal that a gluconeogenic carbon recycling process is occurring at some degree (acetate conversion to malate and this last compound into PEP), since reducing the transformation of ICT into α-KG and the conversion of this metabolite into SUC-CoA (succinyl coenzyme A) induces carbon diversion through the glyoxylate pathway, whose genes were overexpressed. This proposal in turn allows the conservation of two carbon atoms that are not lost as CO_2_ in the transformation of ICT into α-KG and from this last compound into Suc-CoA in the lower section of the TCA cycle, in JM101 grown on glycerol (Table 3, Figure 1).

**Table 5:** Specific growth rates (μ) of strain JM101 cultures grown on glycerol when C4 compounds and amino acids (0.005 g/L) were included in the cultures

**Condition**	**μ** (**h**^**−1**^)
JM101	0.49 (+/–0.01)
JM101 + L-glutamine	0.55 (+/–0.02)
JM101 + L-glutamate	0.48 (+/–0.01)
JM101+ malate	0.57 (+/–0.02)
JM101+ succinate	0.55 (+/–0.02)
JM101+ L-arginine	0.50 (+/–0.01)
JM101+ L-aspartate	0.52 (+/–0.01)
JM101+ L-lysine	0.44 (+/–0.01)
JM101+ L-asparagine	0.50 (+/–0.01)
JM101+ citrate	0.45 (+/–0.01)

### Indole detection, ribonucleoside metabolism and aromatic compounds production capacity in JM101 grown on glycerol

**Figure 6:**
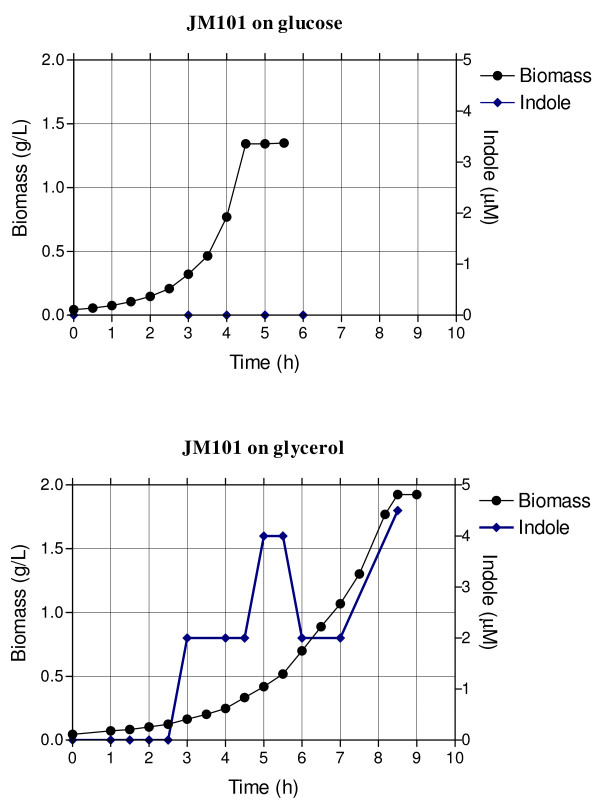
Biomass and indole concentration profiles of strain JM101 grown on glucose or on glycerol.

**Figure 7:**
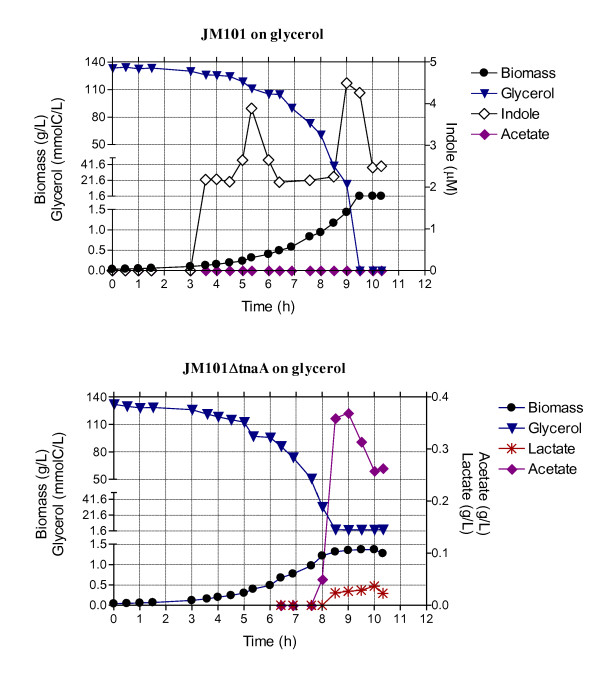
**Glycerol consumption patterns and biomass, indole, acetate and lactate production in strains JM101 and JM101**Δ***tnaA.***

TnaA was one of the proteins highly overproduced (17.03 X) in JM101 grown on glycerol (Table 4). This protein (tryptophan indole-lyase) converts tryptophan into indole, PYR and ammonia (Figure 1). TnaA specific activity was measured at 1 OD and an increment of 400% was found in glycerol compared to growth on glucose (Figure 5). Importantly, micromolar concentrations of indole were detected only during growth on glycerol (Figure 6). Indole production was detected when the biomass concentration was around 0.1 g/L (0.3 OD). Indole production had two peaks; one at 0.31 g/L in the growth curve (0.83 OD) and the other at onset of stationary phase (Figure 6). The JM101Δ*tnaA* derivative with a completely inactivated *tnaA* gene, was evaluated in bioreactors and compared to JM101 during growth on glycerol. No differences in the specific growth rates were detected between the two strains (data not shown). The final biomass concentration was lower for JM101Δ*tnaA* compared to the parental strain (Figure 7). Surprisingly, the JM101Δ*tnaA* derivative produced acetate (0.37 g/L) and small amounts of lactate (0.037 g/L), and as expected no indole was detected (Figure 7). Therefore, the question is what could be the role of indole during the growth of strain JM101 on glycerol? Interestingly, when the first indole peak appeared, extracellular concentration of glycerol was about 3 g/L, indicating that the signal occurred early in the fermentation. The second peak appeared when glycerol was almost exhausted in the media. Therefore, it appears that this signal could be important for continuing exponential growth and to reach a higher biomass at the end of fermentation. It is possible that indole synthesis is part of an adaptive response during different stages of the growth on glycerol cultures. One scenario includes directing the carbon flux into DAHP as a result of the glycerol gluconeogenic metabolism. The synthesis of tryptophan, histidine, NAD and nucleotides (purine and pirymidine) uses 5-phospho-D-ribosyl-α-1-pyrophosphate (PRPP) as a precursor (Figure 1). It appears that about 30–40% of the synthesized PRPP is apparently used for nucleotide synthesis and about 15% to produce tryptophan [63]. It is possible that the continuous indole production from tryptophan enhances PRPP demand and this increases DHAP flux that is diverted through the pentose-phosphate pathway (Figure 1). If tryptophan demand is reduced in the strain lacking TnaA, the flux previously diverted to the pentose pathway could be redirected through glycolysis in this derivative. Under this scenario, the TCA cycle was apparently incapable of coping with the extra flux of AcCoA from PYR and this metabolite was converted to acetate and lactate. A second not excluding scenario that could also be involved in the observed indole response is in agreement with the proposed signal role of this metabolite. It is known that indole increases *crl* expression at both transcriptional and translational levels. In turn, Crl stimulates the activity of RpoS, leading to increased transcription rates of the RpoS regulon in the exponential or stationary phase [41,64]. Mutants in the *crl* or *rpoS* gene exhibited low synthesis levels of FbaB, TalA, PykF, PfkA, GltA and PoxB [41]. Therefore, one of indole roles could be to activate, through Crl, the RpoS activity and finally the genes implied in the balanced distribution of the carbon flux. In these conditions, a lower production of PoxB and TalA whose genes are transcribed by RpoS in the strain JM101Δ*tnaA* could explain the higher glycolytic flux in this derivative (Figure 1). An additional clue about the distribution of the carbon flux in the pentose-phosphate pathway was the overproduction of Cdd (6.97X), Udp (7.41X) and DeoD (2.08X) in JM101 (Table 4). These proteins are involved in the degradation of nucleobases to bases and pentoses. Under PRPP deficient production conditions, the degradation of ribonucleosides is apparently more important than their phosphorylation to nucleotides [63]. Therefore, these data suggest that in JM101 grown on glycerol, the ribose 5-P (R5P) levels are apparently lower as compared to the strain grown on glucose, thus indicating nucleotide recycling during growth on glycerol.

Indole production also suggested that carbon flux into the aromatic amino acids pathway is increased in JM101 grown on glycerol. In agreement, the RT-qPCR values of most of the genes coding for the common aromatic pathway (*aroHaroFaroBaroDaroEaroL*) were slightly overexpressed (Table 3). This capability of strain JM101 to increase carbon flux into the aromatic pathway when grown on glycerol, could explain the high efficiency of production of aromatic compounds of this strain when transformed with plasmid pJLB*aroG*^*fbr*^*tktA* that redirects carbon flux into the aromatic pathway [65-67]. The JM101/pJLB*aroG*^*fbr*^*tktA* derivative grown on mineral media with glycerol as the sole carbon source, produced aromatic compounds with a high yield (Y_aromatics/substrate_) of 0.66 mmolC/mmolC, as compared to the of yield of 0.07 mmolC/mmolC that was obtained when this derivative was grown on glucose as the sole carbon source (Table 6).

**Table 6:** **Aromatic compounds yields of strains JM101 and JM101/pJLB*aroG***^***fbr***^***tktA*in flask cultures with glucose or glycerol as sole carbon sources**

Strains	Carbon source	Y_arom_(mmolC/mmolC)
JM101	Glucose	Not detected
JM101	Glycerol	0.25^−3^ (+/–0.20^−4^)
JM101/pJLB*aroG*^*fbr*^*tktA*	Glucose	0.07 (+/–0.002)
JM101/pJLB*aroG*^*fbr*^*tktA*	Glycerol	0.66 (+/–0.005)

(See Methods).

## Conclusions

Overexpression of the genes and overproduction of their coded proteins involved in glycerol uptake and metabolism were detected. These proteins are responsible for the transport and incorporation of glycerol as DHAP, one of the metabolites of the glycolytic pathway. Overexpression of several glycolytic and gluconeogenic genes in the upper part of the glycolytic pathway, especially *fbaA*, *fbaB*, *fbp* and *pgi*, are responsible for the production of G6P from DHAP. Low F1,6P/F6P levels could be the signal for the induction of some of these regulons when strain JM101 is growing on glycerol. This phenomena is reinforced by the differential expression of some genes regulated by Cra which respond to low F1,6P concentrations.

The detected overexpression of the *mal*/*lam* and *mgl*/*gal* regulons and the overproduction of their coded proteins and some genes regulated by RpoS, indicate that JM101 apparently induced a “carbon stress and carbon scavenging response” when growing on glycerol as the sole carbon source, indicating as reported that this carbohydrate is a poor carbon source. This proposition is in agreement with the involvement of RpoS, the master regulator of stress response in glycerol fermentation, since its inactivation reduced 10% the μ and delayed by two hours the growth of JM101Δ*rpoS*.

The detected overexpression of *poxB*, *acs*, *pta*, *actP*, *acnB* and the glyoxylate shunt genes (*aceBA* and *glcB*), some of them transcribed by RpoS, indicates that JM101 is apparently producing and simultaneously consuming acetate when growing on glycerol as the sole carbon source. In agreement with this proposal, was the result that no acetate was detected when growing on glycerol, and acetate can be coutilized with glycerol as carbon sources. It has been proposed that when *E. coli* is growing slowly on glucose apparently reduces the carbon flux through the Pdh system, which yields AcCoA directly from PYR, and diverts part of the carbon flux via PoxB that synthesizes acetate from PYR, with a concomitant reduction of quinones at the membrane. Acetate, is then utilized by Acs and transformed into AcCoA, apparently creating a “carbon acetate recycling” mechanism which is also apparently present in PB11 (a derivative of JM101 lacking PTS) that grows slowly on glucose. Therefore, it appears that in addition to the glycolytic metabolism that is functioning in JM101 when growing on glycerol, carbon scavenging responses are also observed in this strain when it grows on this poor carbon source. Consistent with this proposed metabolic response, in JM101 cultures grown on glycerol as mentioned, no acetate was detected, because this strain probably recycles acetate through the PoxB-Acs-glyoxylate shunt enzymes and is capable of coutilizing glycerol and acetate. In accordance, JM101 derivatives with inactive *poxB* or *pckA* genes accumulated acetate and their specific growth rates were affected. The induction of this mechanism apparently permitted a more efficient carbon utilization and acetate recycling in these growing conditions. In agreement, the downregulation of *icd* and *lpdA* coding for IcdA and LpdA (part of the SucABCD complex), supports the proposition that the carbon flux is reduced through the lower section of the TCA cycle, thus enabling carbon gluconeogenic recycling through the glyoxylate shunt, since *aceBA* and *glcB* were overexpressed. As a result, if this hypothesis is correct, less carbon should be lost as CO2 in the lower section of the TCA pathway.

In agreement with a reduced TCA cycle during the growth of JM101 on glycerol, it appears that relatively low production of C4 carbon metabolites occurred, given that the μ of this strain was enhanced in cultures grown on glycerol when succinate, malate or aminoacids derived from 2-oxoglutarate were added to the fermentation. This supports the hypothesis that when *E. coli* grows slowly, part of the carbon is recycled, preserved through the glyoxylate shunt and not lost as CO2 in the TCA cycle.

Indole production in JM101 grown on glycerol indicates an important carbon flux through the aromatic amino acids pathway. Indole is a signaling molecule that activates Crl for modulating the expression of certain RpoS-Crl regulons; however, a signaling role of this metabolite is not completely clear at the moment. It has been proposed that the expression of *rpoS* is not only negatively controlled by cAMP-CRP high levels but also inversely correlated with growth rate. Since glycerol is a relatively poor carbon source, intracellular high levels of cAMP-CRP are expected in JM101. Indole synthesis could stimulate RpoS activity under these non favorable growth conditions. Additional studies should be conducted to gain a better understanding into the role of this signal. Nevertheless, the detected overproduction of Cdd, DeoD, and Upp suggests that the carbon flux through the pentose-phosphate pathway is reduced when glycerol is used as the sole carbon source as compared to the flux when glucose is utilized. It appears that when glycerol is used as the only carbon source, a carbon stress mechanism occurs. In this condition, RpoS regulons could be also indirectly activated by indole, allowing a more adequate response to growth on carbon limited conditions. Importantly, and in agreement with an increased carbon flux through the aromatic pathway growing on glycerol when JM101 is transformed with plasmid pJLB*aroG*^*fbr*^*tktA* that redirects and enhances carbon flux into the aromatic pathway, this strain showed a yield increase of aromatic compounds almost 9-fold as compared to the production of these metabolites when glucose is used as the sole carbon source.

The transcription levels of most of the measured genes correlated with the detected values of the proteins produced in the analyzed growth conditions, using glycerol as the only carbon source. Also, the specific activities of various measured proteins correlated with these values.

In this contribution we described new features of *E. coli* physiology during the growth on glycerol, as detected through a proteomic-transcriptional study and kinetic-stoichiometric evaluation of strain JM101 and some isogenic mutants in certain key PEP-PYR genes (*poxB*, *ppc*, *pckA*, *pykA* and *pykF*) and in *rpoS*. It appears that when glycerol is used as the sole carbon source in addition to the glycolytic metabolism, a carbon stress response occurs that includes carbon scavenging and acetate gluconeogenic carbon recycling responses mediated mainly by RpoS. In addition, this regulator could also be activated by Crl through indole, allowing a more adequate response to growth on glycerol, a carbon limited condition. The simultaneous utilization of various metabolic redundant alternative mechanisms when growing on glycerol indicates metabolic plasticity of *E. coli*. Understanding these capacities advances the knowledge on the physiological responses *E. coli* is capable of, and enhances our capacities for developing more advanced metabolic engineering strategies using this bacterium for the production of specific metabolites.

## Methods

### Bacterial strains

Strain JM101 [23,67] and derivatives with specific inactivated genes used in this work are listed in Table 7.

**Table 7:** Strains used in this report

**Strains**	**Relevant characteristics**	**Source**
*E. coli* JM101	F’ *tra*D36 *proA* + *proB* + *lac*Iq *lac*Z_M15/*supE thi* _(*lac*-*proAB*)	[23,67]
JM101Δ*galP*	JM101*galP::tc*	This work
JM101Δ*pck*	JM101*pck::cat*	This work
JM101Δ*ppc*	JM101*ppc::cat*	This work
JM101Δ*pykA*	JM101*pykA::cat*	This work
JM101Δ*pykF*	JM101*pykF::cat*	This work
JM101Δ*tnaA*	JM101*tnaA::cat*	This work
JM101Δ*poxB*	JM101*poxB::cat*	[19]
JM101Δ*rpoS*	JM101*rpoS::tc*	[68]
JM101/pJLB*aroG*^*fbr*^*tktA*		[65,66]

### Genetic procedures and recombinant DNA techniques

PCR reactions were performed using Platinum Taq polymerase accordingly to the manufacturer’s recommendations (Invitrogen, USA). Complete inactivation of *pcktnaApykApykF* and *ppc* genes was performed by transduction, using P1*vir* phage grown on strains from the Keio collection [69] carrying these genes inactivated by the Datsenko method [70]. Gene inactivations were confirmed using PCR reactions with specific oligonucleotides (data not shown). Table 8 includes the oligonucleotides utilized in this report.

**Table 8:** Oligonucleotides utilized in this report (gene inactivations and RT-qPCR)

A. Oligonucleotides used for the detection of gene inactivations		
*pckA*	pckAFw	CAG GAA TGC GAT TCC ACT CA
	pckARv	GTG CAG CGT ATC GTG GAT AA
*ppc*	ppcFw	GCA TCT TAT CCG ACC TAC AC
	ppcRv	GCC TGT AGC AGA GTA GAG AA
*pykA*	pykAFw	CTG AAG GAA TCG CGT CGT TTT GA
	pykARv	CGG CGG ATG AAT GAA GAA
*pykF*	pykFor	ACA AGC ACA CAT TCC TCT GCA
	pykRev	AAA ACA GGA TGC TTC CAT CG
*tnaA*	tnaAFw	TTC TGT AGC CAT CAC CAG AG
	tnaARv	CCG GCA AGA TCA ACA GGT AA
*galP*	galpAa	CAT GTA TTA CGC GCC GAA AA
	galpAb	TGG CAA GTA CGT TGG TCA GG
B. Oligonucleotides used for RT-qPCR assays*		
*cpdA*	cpdAa	CAG CAT TTC GCT GAA GGC AT
	cpdaB	GCA TCC TGT AAC GCG CTG TAC
*glpD*	glpDa	ATG GTG CTG GTA TCG CGG
	glpDb	TTT TGA ACT GGC GGA AGA GG
*glpF*	glpFa	AGG CCA GTG CAT TGC TGA AT
	glpFb	ACT GAC CAA AAG ACG CAC CAG
*glpK*	glpKa	CTC GAC CAT GTG GAA GGC TC
	glpKb	ACA CGG CCC TGA GTC ATT TT
*pntA*	pntAa	AAC CAG CGC CGA AGC TAA TT
	pntAb	GTA TTC ACA GTT GCC GCC GT
*udhA*	udhAa	AAG GCT GTG ACG ATG GTG TG
	udhAb	CGA ATC GGT ATT ACC GGT GC

* Oligonucleotide sequences of the remaining utilized genes in Table 3 have been published elsewhere [12,13,19,20,68].

### Growth conditions

#### Batch cultures

M9 medium, containing (per liter): 6 g Na_2_HPO_4_; 3 g KH_2_PO_4_; 0.5 g NaCl; 1 g NH_4_Cl; 2 mM MgSO_4_; 0.1 mM CaCl_2_; 0.01 g Vit B1, and 2 g/L glucose, was utilized for growing the fermentor inocula. A higher concentration of glucose or glycerol (4 g/L, approximately 130 mmolC/L, depending on the molecular weight of the carbon source) was utilized in the bioreactors or in 500 mL shake flask studies, when only one carbon source was employed. When two carbon sources were used, the same amount of each carbon source (2 g/L, approximately 65 mmolC/L) was employed. When acetate was added as carbon source, the concentration employed was 0.33 g/L (approximately 11 mmolC/L). Derivative JM101/pJLB*aroG*^*fbr*^*tktA*[65,66] was also grown in 500 mL shake flasks using glycerol or glucose as sole carbon sources. IPTG (0.1 mM) was added at the beginning of the fermentation. Tetracycline (30 μg/mL) was included in the medium for plasmid maintenance. Samples from these cultures were obtained during the whole fermentation process. Cells were centrifuged and analyzed for the production of aromatic compounds as described below.

#### Bioreactor conditions

Strain JM101 was cultivated in a 1 L bioreactor (Applikon Biotechnology, Netherlands) with a working volume of 0.75 L, 600 rpm, pH controlled at 7 with NH_4_OH (2.0%), and air flow rate of 1 vvm, starting at an OD_600_ of 0.10 and collected when growing in the log phase at an OD_600_ of 1.

### Kinetic and stoichiometric parameters

Data represent the average of at least three different cultures. Cell growth was measured by monitoring the optical density at 600 nm (OD_600_) in a spectrophotometer (Beckman DU700). OD_600_ was converted into dry cellular weight (biomass concentration) using a standard curve (1 OD_600_ = 0.37 g/L of dry cellular weight). Specific growth rates (μ) were determined by fitting the biomass data versus time to exponential regressions. The biomass yield (Yx/s) was estimated as the coefficient of linear regression of biomass concentration versus substrate concentration of glucose and glycerol, in grams of biomass/mmolC of substrate. The specific carbon consumption rate (q_s_) was determined as the ratio of μ to Y_x/s_, according to Monod’s model reported elsewhere [19,71]. The aromatic compounds yield presented in Table 6 was estimated as the sum of the total production of 3-deoxy-D-arabinoheptulosonate-7-phosphate (DAHP), dehydroshikimate (DHS), shikimate (SHIK) and indole in mmolC divided by the total carbon source consumed in mmolC of substrate. L-tryptophan, L-phenylalanine and L-tyrosine were not detected in these fermentations.

### Analytical methods

Metabolite concentrations were determined with an HPLC system (600E quaternary bomb, 717 automatic injector, 2410 refraction index, and 996 photodiode array detectors (Waters, USA). An Aminex HPX-87 H column (300 by 7.8 mm; 9 Am) (Bio-Rad Laboratories, USA) was used. Running conditions were: mobile phase, 5 mM H_2_SO_4_; flow, 0.5 mL/min, and temperature, 50°C. Under these conditions, D-glucose, D-glycerol, DAHP, DHS, SHK, acetate and lactate were detected by refraction index [71]. Indole and the aromatic aminoacids L-tryptophan, L-phenylalanine and L-tyrosine in culture supernatants were quantified using an Agilent 1100 high-performance liquid chromatography system (Agilent Technologies, USA) equipped with a Phenomenex Synergy Hydro RP18 column (150 by 4.6 mm; 4 μm) attached to an Agilent 1100 electrospray mass spectrometry detection system (Agilent Technologies, USA) [72]. Samples were eluted with 10% methanol in 0.1% acetic acid in water at an isocratic flow rate of 0.5 mL/min. UV detection was performed at 220 nm. A dual solvent system, at a column flow rate of 1.0 mL/min, was used for separation [73]. Solvent A consisted of 0.1% trifluoroacetic acid in water, while solvent B was 0.1% trifluoroacetic acid in acetonitrile. Starting conditions were: 95% solvent A and 5% solvent B, the solvent gradient was run for 8 min and ended at 20% solvent A and 80% solvent B. From minutes 8–10, the ratio was maintained at 20% solvent A and 80% solvent B. From minutes 10 to 15, the ratio was 95% solvent A and 5% solvent B.

### RNA extraction, DNAse treatment of RNA and cDNA synthesis for RT-qPCR analysis

#### Sample management and treatment

Strain JM101 was grown in different bioreactors using glycerol or glucose (as the control) as carbon sources. After the bioreactor was inoculated at the same optical density (0.1), the culture was monitored to verify and reproduce the μ, q_s_ and Y_x/s_ values. When the fermentation reached 1 OD, 7 mL samples were taken directly from the bioreactor using a 1 mm diameter pipe and collected in 15 mL cap tubes containing 2 mL of RNA protect Bacteria Reagent (Quiagen^TM^, Netherlands) and mixed carefully. After 1 min, the samples were centrifuged at 8,000 rpm for 8 min. The pellet was immediately frozen at −70°C until RNA extraction.

#### Nucleic acid extraction

Total RNA was isolated and purified using the hot-phenol method, with some modifications. Samples containing 7 mL of the collected frozen cells were resuspended in 1 mL buffer I (0.3 M sucrose, 0.1 M sodium acetate), treated with 20 μL lysozyme (10 mg/mL in TE buffer) and incubated for 10 min at room temperature. 2 mL buffer II (0.01 M sodium acetate, 2% SDS) were added and the mixture was incubated for 3 min at 65°C. The lysate was extracted with 2 mL of hot phenol and heated for 3 min at 65°C. A second extraction with hot phenol was performed without heating the mixtures. Samples were then extracted with 2 mL of a phenol:chloroform mixture (1:1), precipitated with 0.1 volume of 3 M sodium acetate (pH, 5.2) and 2.5 volume of ethanol and centrifuged for 15 min at 4°C, 10,000 rpm. Samples were then suspended in a volume containing 300 μL of DNAse and RNAse-free water (Ambion Inc, USA) with RNAse inhibitor (Thermo-Scientific, USA) and extracted twice with 1 volume of chloroform. Finally, samples were precipitated as before and suspended in 300 μL TE buffer (Ambion Inc, USA). RNA was analyzed on formaldehyde agarose gel for integrity. RNA concentrations were quantified using Nanodrop 2000c (Thermo Scientific, USA); the 260/280 and 260/230 ratios were examined for protein and solvent contamination. For all samples, the 260/280 nm absorbance values were between 1.9–2.0 and in the range of 2.0–2.3 for the 260/230 nm ratio. RNA samples were stored at −70°C. For DNAse treatment, total RNA samples were treated with Turbo DNA-free kit (Ambion Inc, USA) at 37°C for 30 min, following manufacturer’s instructions. To determine whether RNA samples were significantly contaminated with genomic DNA, samples were subjected to conventional PCR with primers for the *arcA* gene [12,13]. Since these primers were designed to recognize genomic DNA, the presence of a detectable PCR product on an ethidium bromide-stained agarose gel would indicate that the specific RNA sample was contaminated with genomic DNA. PCR reactions were performed with Taq polymerase (Thermo-Scientific, USA). The cycling parameters were: 95°C for 5 min; 30 cycles at 95°C for 1 min, 55°C for 1 min and 72°C for 1 min, plus an extension step at 72°C for 5 min. Additionally, DNAse-treated RNA samples were used for RT-qPCR analysis of the same *arcA* gene, using the appropriate oligonucleotides *arcA*a (forward) and *arcA*b (reverse)] [12,13]. As in the PCR case, all utilized samples did not produce a 101 bp amplimer, indicating that small fragments of genomic DNA contaminating the samples were not present. cDNA was synthesized using RevertAid^TM^ H minus first strand cDNA synthesis kit and following the manufacturer’s conditions (Thermo-Scientific., USA). For each reaction, approximately 5 μg of RNA and a mixture of 10 pmol/μL of specific DNA reverse primers (b primers) for each measured gene were used. Nucleotide sequences of these genes have been previously published [12,13,24,72] or are listed in Table 8. cDNA were used as template for RT-qPCR assays.

#### RT-qPCR

RT-qPCR was performed with the ABI Prism 7000 Sequence Detection System and 7300 Real Time PCR System (Perkin Elmer/Applied Biosystems, USA) using the Maxima^R^ SYBR Green/ROX qPCR Master Mix (2X) kit (Thermo-Scientific, USA). MicroAmp Optica 96-well reaction plates (Applied Biosystems, USA) and Plate Max ultraclear sealing films (Axygen Biosciences, USA) were used in these experiments. Amplification conditions were 10 min at 95°C, followed by a two-step cycle at 95°C for 15 s and 60°C for 60 s for a total of 40 cycles, to finish with a dissociation protocol (95°C for 15 s, 60°C for 1 min, 95°C for 15 s and 60°C for 15 s). DNA sequences of the primers for specific amplifications were designed using the Primer Express software (Applied Biosystems, USA). Some of these have been previously published [12,13,24] and the rest of the sequences are included in Table 8. All RT-qPCR experiments complied with the MIQE guidelines (Minimum Information for Publication of Quantitative Real-Time PCR Experiments) [74,75]. The length of all the utilized oligonucleotides (forward and reverse), was between 18 and 21 nucleotides, with GC% between 45 to 60 and Tm between 58 to 60°C. The size of all amplimers was 101 bp. The final primer concentration was 0.2 μM in a total volume of 12 μL. Five ng of target cDNA for each gene were added to the reaction mixture, since higher cDNA concentrations (>10 ng) are not in the dynamic range of the reference gene *ihfB* (see below). Hence the obtained values cannot be correctly normalized for this higher cDNA concentration. All experiments were performed at least in triplicate for each gene of each strain, obtaining very similar values (differences <0.3 SD). A non-template control reaction mixture was included for each gene and values appeared for all genes, after cycle 31. Standard curves were built to evaluate PCR efficiency and all the genes had R^2^ values above 0.9976 with slopes between −3.4 to–3.7. The quantification technique used to analyze data was the 2^−ΔΔCq^ method described by Livak and Schmittgen [76]. Data were normalized using the *ihfB* gene as an internal control (reference gene). The same reproducible expression level of this gene was detected in all the strains in the conditions in which bacteria were grown and analyzed; this is the most important characteristic that a reference gene should have in accordance with the MIQE guidelines. Supporting information (Additional file 2) presents the *ihfB* gene values detected for the utilized strains. These results demonstrate the stability of the expression of this reference gene in all the analyzed derivatives for the conditions used in this report and also on previous reports utilizing these strains and other derivatives [12,13,24,74]. For each analyzed gene in all strains, the transcription level of the strain JM101 was considered equal to one, and it was used as control to normalize the data. Therefore, data are reported as relative expression levels, compared to the expression level of the same gene in strain JM101. The results presented in Table 3 are the averages of at least three independent measurements of the RT-qPCR expression values for each gene. Values were obtained from different cDNAs generated from at least five independent bioreactor samples [13].

### Enzymatic assays

For each enzymatic assay, approximately 15 mL of cultures were harvested and centrifuged at 10,000 rpm, 4°C for 1 min. The pellets were stored at −20°C until the enzymatic assay was performed (not further than two days). Before each assay, the pellets were dissolved in their corresponding enzyme specific buffers (see below). Dissolved cells were disrupted by three sonication steps with 20 s intervals at 14 milliohms. Cell debris was removed by centrifugation at 10,000 rpm, 4°C for 10 min. Enzymatic assays were performed at 30°C using a Thermo Spectronic-Biomate spectrophotometer. The buffer and substrates were mixed in a spectrophotometric cuvette to a final reaction volume of 1 mL. The wavelength and millimolar extinction coefficients for NADH_2_, NADP^+^ and NADPH_2_ were 340 nm and 6.22 cm^−1^ mM^−1^, respectively. For phenylhydrazine-HCl, the wavelength and millimolar extinction coefficients were 324 nm and 16.8 cm^−1^ mM^−1^, respectively. One unit of specific enzyme activity (U) was defined as the amount of enzyme required to convert 1 mole of substrate into the specific product per minute per milligram of protein. The amount of protein was measured by the Bradford method with bovine serum albumin as the standard [77]. Utilized buffers: Pyruvate kinase (Pyk): 100 mM Tris–HCl (pH, 7.5), 5 mM ADP, 1 mM DTT, 10 mM KCl, 15 mM MgCl_2_, 0.5 mM phosphoenolpyruvate (PEP), 0.25 mM NADH_2_, 10 U lactate dehydrogenase (Ldh) [78]. PEP carboxylase (Ppc): 60 mM Tris–HCl (pH, 9.0), 10 mM MgCl_2_, 10 mM NaHCO_3_, 0.15 mM NADH_2_, 5 mM PEP and 2U of malate dehydrogenase (Mdh) [79]. PEP carboxykinase (PckA): 10 mM TES Buffer (pH, 6.6), 10 mM MgCl_2_, 5 mM MnCl_2_, 1 mM DTT, 10 mM ADP, 75 mM NaHCO_3_, 0.3 mM NADH_2_ and 20 U of Mdh; in this case one must add cell extract, incubate at 37°C for 15 min, then add 10 mM PEP to start the reaction [79]. Malic enzyme (MaeB): 100 mM Tris–HCl (pH, 7.8), 5 mM MgCl_2_, 0.6 mM NADP^+^, 40 mM malate [79]. Isocitrate lyase (Icl): 50 mM morpholipepropanesulfonic acid (MOPS; pH, 7.3), 1 mM EDTA, 5 mM MgCl_2_, 4 mM phenylhydrazine HCl (FH) and 12.5 mM L-isocitrate [80]. Isocitrate dehydrogenase (Icdh): 50 mM phosphate buffer (pH 7.5), 5 mM MgCl_2_, 2 mM NADP^+^, 2.5 mM D,L isocitrate [81]. Tryptophanase (TnaA): 1000 mM potassium phosphate (pH, 8.3), 0.81 mM pyridoxal 5-phosphate, 50 mM L-tryptophan (pH 10.8), trichloroacetic acid 6.1 N, toluene, p-dimethylaminobenzaldehyde solution 5%(w/v), hydrochloric acid-alcohol 895 mM [82].

### Proteomic analysis

Protein extraction and two-dimensional gel electrophoresis were carried out as previously described [83]. Gels were dyed in colloidal Coomassie [84] and scanned in a GS-800 densitometer (Bio-Rad Laboratories, CA). Digital images were analyzed and compared using the PDQuest 8.0.1 software from the same company. Each experiment was done in triplicate. Only reproducible phenotypes, with a Student’s *t* test value p ≤ 0.05 are shown. Additional file 3 contains other important proteomic parameters. Once the digital image of each gel was compared against the rest, the electrophoretic entities of interest were cut, alkylated, reduced, digested and automatically transferred to a MALDI analysis target by a Proteineer SP II and SP robot using the SPcontrol 3.1.48.0 v software (Bruker Daltonics, Germany), with the aid of a DP Chemicals 96 gel digestion kit (Bruker Daltonics, Germany) and processed in a MALDI-TOF Autoflex (Bruker Daltonics, Germany) to obtain a mass fingerprint. One hundred satisfactory shots were performed in 20 shotsteps; the peak resolution threshold was set at 1500, the signal/noise ratio of tolerance was 6, and contaminants were not excluded. The spectrum was annotated by the flexAnalysis 1.2 v SD1 Patch 2 (Bruker Daltonics, Germany). The search engine MASCOT [85] was used to compare the fingerprints against the UNIPROT [86] release 2011–01 database with the following parameters: Taxon- *Escherichia coli*, mass tolerance of up to 200 ppm, one miss-cleavage allowed. Carbamidomethyl was the fixed modification and oxidation of methionine the variable modification.

## Competing interests

The authors declare that they have no competing interests.

## Authors’ contributions

KMG and FB designed the experiments, analyzed the results and wrote the manuscript. KMG carried out the fermentations, samples analysis, gene inactivations, TnaA assay, RNA extraction and samples preparations for proteomic experiments. NF performed RNA purification, cDNA synthesis, RT-qPCR experiments and data processing. HMCA carried out Pyk and JM101/pJLB*aroG*^*fbr*^*tktA* fermentations, the enzymatic assays, DAHP semipurification. GMB performed the 2-D electrophoresis gel and MALDI-TOF analyses of proteins. GH performed the technical support for DAHP semipurification and HPLC determinations. SE, GG and OTR critically revised the results and the manuscript. All the authors have read and approved the publication of the manuscript.

## Supplementary Material

Additional file 1 Central metabolic genes overexpressed or underexpressed during growth on glycerol as compared to glucose. Certain regulators involved in the expression of these genes are also included. Click here for file

Additional file 2 This figure includes the positions of the amplification curves for the *ihfB* gene and the Ct values of this gene (see Methods), in the different strains employed in this study. As can be seen, all the amplification curves of the *ihfB* gene, which has been used as the reference gene, show very similar values. The values presented in the table are from five different fermentations of each strain. These results demonstrate that the same reproducible expression levels are obtained for the *ihfB* gene in all strains. This is the most important characteristic that a reference gene should have in accordance with the MIQE guidelines [13,74]. These results corroborate the stability of the expression of the reference *ihfB* gene in these strains under the utilized conditions. Click here for file

Additional file 3 This file contains important proteomic parameters for the identified proteins (Gi, Score, Mw, pI, %cov, EC number and gene ID). Click here for file
