# Supplementary material for: New insights into Escherichia coli metabolism: carbon scavenging, acetate metabolism and carbon recycling responses during growth on glycerol
Source: Microb Cell Fact. 2012 Jul 4;11:46. doi: 10.1186/1475-2859-11-46 (PMC3390287; doi:10.1186/1475-2859-11-46)
Supplement: Additional file 1 — Central metabolic genes overexpressed or underexpressed during growth on glycerol as compared to glucose. Certain regulators involved in the expression of these genes are also included. [file 1475-2859-11-46-S1.doc]

**Additional file 1**

**Central metabolic genes overexpresed or underexpressed during grown on glycerol. Certain regulators which are involved in the expression of these genes are also included.**

| Gene name | 2^-ΔΔCT^ | **Rpos** | **FruR** | **Crp** | **ArcA** | **FNR** |
| --- | --- | --- | --- | --- | --- | --- |
| ***aceA*** | 1.97+0.39 |  | (+) | (-) | (-) |  |
| ***aceB*** | 1.95+0.06 |  | (+) | (-) | (-) |  |
| ***aceE*** | 0.67+0.17 | (-) |  | (+) | (-) |  |
| ***acnA*** | 1.58+0.26 | (+) | (+) | (+) | (-) |  |
| ***acnB*** | 4.71+0.47 |  | (-) | (+) | (-) |  |
| ***acs*** | 4.16+0.34 | (+) |  | (+) |  |  |
| ***actP*** | 13.49+1.31 | (+) |  | (+) |  |  |
| ***aroG*** | 0.50+0.13 |  |  |  |  |  |
| ***eda*** | 1.83+0.29 |  | (-) |  |  |  |
| ***edd*** | 4.45+0.29 |  | (-) |  |  |  |
| ***fbaA*** | 1.52+0.09 | (+) | (-) | (+) |  |  |
| ***fbaB*** | 2.76+0.22 | (+) |  |  |  |  |
| ***frdA*** |  |  |  |  |  | (+) |
| ***frdB*** |  |  |  |  |  | (+) |
| ***frdC*** |  |  |  |  |  | (+) |
| ***frdD*** |  |  |  |  |  | (+) |
| ***fumA*** | 2.44+0.45 |  | (-) | (+) |  |  |
| ***fumB*** | 7.49+0.40 |  |  | (+) | (+) |  |
| ***gapC-2*** | 3.19+0.67 |  |  |  |  | (+) |
| ***glcB*** | 1.81+0.56 |  |  |  | (-) |  |
| ***glk*** | 3.81+0.14 | (+) | (-) |  |  |  |
| ***glpF*** |  |  |  | (+) |  |  |
| ***glpK*** |  |  |  | (+) |  |  |
| ***glpD*** |  |  |  | (+) | (-) |  |
| ***lpdA*** | 0.56+0.05 | (+) |  | dual | (-) |  |
| ***mdh*** |  |  |  | (+) | (-) |  |
| ***ompC*** |  |  | (+) |  |  |  |
| ***pckA*** | 5.56+1.77 | (+) | (-) |  |  |  |
| ***pfkA*** | 1.91+0.22 | (+) |  |  |  |  |
| ***pfkB*** | 2.15+0.01 | (+) |  |  |  |  |
| ***pgi*** | 1.58+0.37 | (+) |  |  |  |  |
| ***poxB*** | 7.33+0.56 | (+) |  |  |  |  |
| ***pta*** | 2.10+0.23 |  |  |  |  | Not know |
| ***talA*** |  | (+) |  |  |  |  |
