# Supplementary figures and images for: New insights into Escherichia coli metabolism: carbon scavenging, acetate metabolism and carbon recycling responses during growth on glycerol
Source: Microb Cell Fact. 2012 Jul 4;11:46. doi: 10.1186/1475-2859-11-46 (PMC3390287; doi:10.1186/1475-2859-11-46)

**Additional file 2**

**
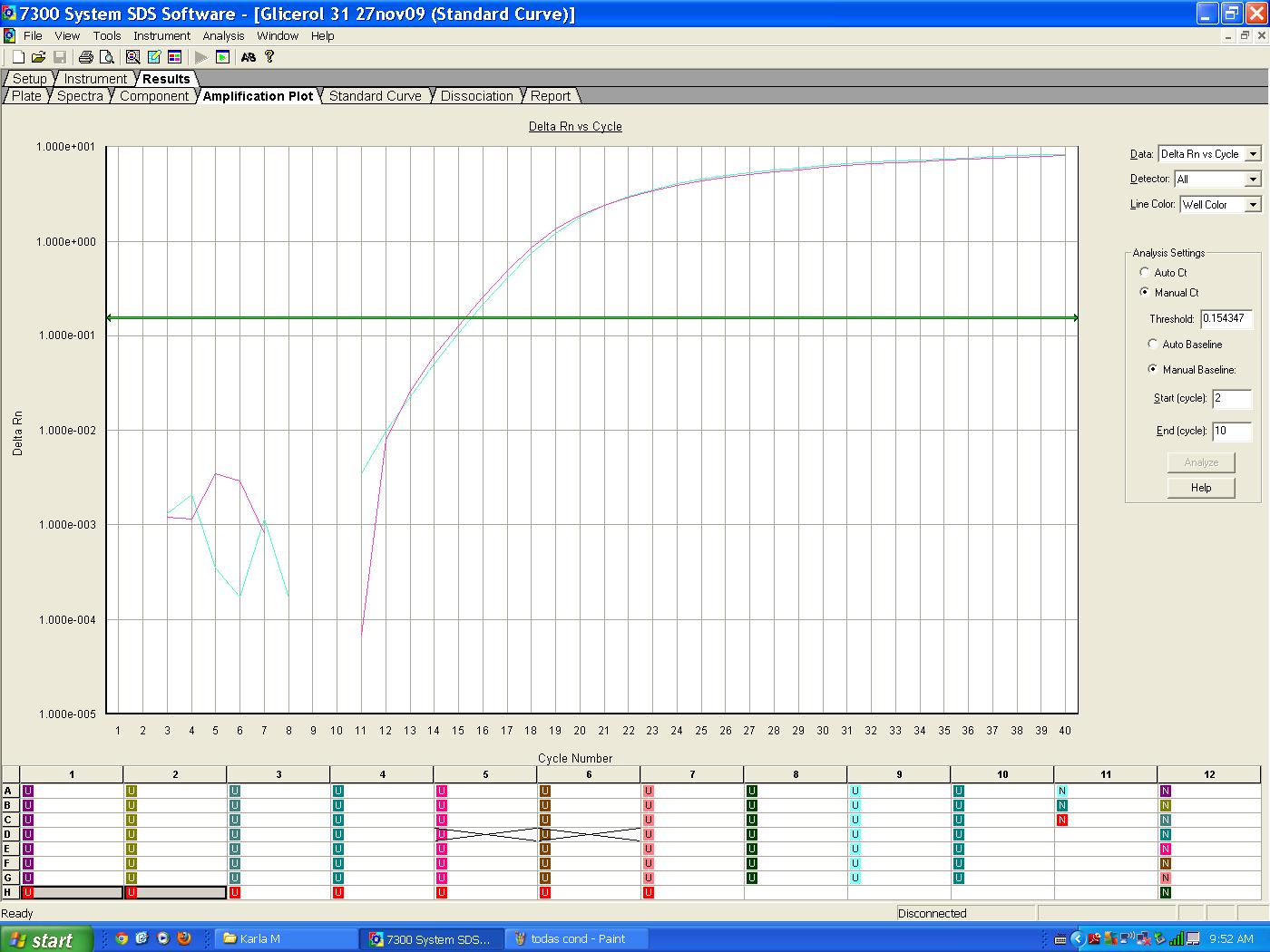
**

| **Strain JM101 growth conditions** | **Cq** |
| --- | --- |
| Glucose | 17.18 |
| Glycerol | 17.33 |

Supplement: Additional file 2 — This figure includes the positions of the amplification curves for the ihfB gene and the Ct values of this gene (see Methods), in the different strains employed in this study. As can be seen, all the amplification curves of the ihfB gene, which has been used as the reference gene, show very similar values. The values presented in the table are from five different fermentations of each strain. These results demonstrate that the same reproducible expression levels are obtained for the ihfB gene in all strains. This is the most important characteristic that a reference gene should have in accordance with the MIQE guidelines [13,74]. These results corroborate the stability of the expression of the reference ihfB gene in these strains under the utilized conditions. [file 1475-2859-11-46-S2.doc]
